# The effect of a multi-faceted quality improvement program on paramedic intubation success in the critical care transport environment: a before-and-after study

**DOI:** 10.1186/s13049-023-01074-0

**Published:** 2023-02-22

**Authors:** Johannes von Vopelius-Feldt, Michael Peddle, Joel Lockwood, Sameer Mal, Bruce Sawadsky, Wayde Diamond, Tara Williams, Brad Baumber, Rob Van Houwelingen, Brodie Nolan

**Affiliations:** 1 Ornge, 5310 Explorer Drive, Mississauga, ON L4W 5H8 Canada; 2 Department of Emergency Medicine, St. Michael’s Hospital Toronto, 36 Queen St East, Toronto, ON M5B 1W8 Canada; 3 Department of Emergency Medicine, London Health Sciences Centre, 800 Commissioners Drive, London, ON N6A 5W9 Canada; 4 Department of Emergency Medicine, Sunnybrook Health Sciences Centre, 2075 Bayview Ave, Toronto, ON M4N 3M5 Canada

## Abstract

**Introduction:**

Endotracheal intubation (ETI) is an infrequent but key component of prehospital and retrieval medicine. Common measures of quality of ETI are the first pass success rates (FPS) and ETI on the first attempt without occurrence of hypoxia or hypotension (DASH-1A). We present the results of a multi-faceted quality improvement program (QIP) on paramedic FPS and DASH-1A rates in a large regional critical care transport organization.

**Methods:**

We conducted a retrospective database analysis, comparing FPS and DASH-1A rates before and after implementation of the QIP. We included all patients undergoing advanced airway management with a first strategy of ETI during the time period from January 2016 to December 2021.

**Results:**

484 patients met the inclusion criteria during the study period. Overall, the first pass intubation success (FPS) rate was 72% (350/484). There was an increase in FPS from the pre-intervention period (60%, 86/144) to the post-intervention period (86%, 148/173), *p* < 0.001. DASH-1A success rates improved from 45% (55/122) during the pre-intervention period to 55% (84/153) but this difference did not meet pre-defined statistical significance (*p* = 0.1). On univariate analysis, factors associated with improved FPS rates were the use of video-laryngoscope (VL), neuromuscular blockage, and intubation inside a healthcare facility.

**Conclusions:**

A multi-faceted advanced airway management QIP resulted in increased FPS intubation rates and a non-significant improvement in DASH-1A rates. A combination of modern equipment, targeted training, standardization and ongoing clinical governance is required to achieve and maintain safe intubation by paramedics in the prehospital and retrieval environment.

## Background

Endotracheal intubation (ETI) is a high-risk procedure performed by physician and paramedics in the austere prehospital and retrieval environment [1–4]. While ETI is a key component of care for most prehospital critical care or retrieval services, research findings have been equivocal, with suggestions of benefit, no difference, or even harm [4–7]. A number of factors are likely responsible for this wide range of findings of patient outcomes following prehospital ETI. Careful patient selection is a key component to assure clinical benefit and should include the indication, resources available, likelihood of complications, and anticipated transport time and modality [2, 8]. Once the decision for prehospital ETI has been made the procedure needs to be undertaken to a high standard if it is to be beneficial [9]. The quality of ETI is difficult to measure [10], particularly if facilitated by sedative drugs (drug facilitated intubation—DFI) or through a combination of sedative drugs and a paralytic drug (rapid sequence intubation—RSI) [11]. Common variables reported as proxy-measures for the quality of ETI during prehospital DFI or RSI are the first pass success (FPS) rates, the number of intubations attempts required, overall failure rates of intubation (ie. ETI aborted after usually multiple attempts), and the incidence of new hypoxia or hypotension during DFI or RSI [4, 12–14]. A both process- and patient-focused quality metric suggested by the international Ground and Air Medical Quality in Transport organization (GAMUT) is DASH-1A: successful intubation on the first attempt, without the occurrence of hypoxia or hypotension [14]. Many prehospital critical care and retrieval services have published improved quality of prehospital ETI provided, as measured by these variables. Factors associated with improved quality in previous publications are the use of checklists and standard operating procedures, the introduction of video laryngoscopes (VLs), simulation training, and audit and review of cases [10, 15–17].

In the past, internal quality control measures demonstrated a wide variability of FPS rates for paramedics undertaking intubations within our large provincial Canadian critical care transport organization. This study describes the FPS and DASH-1A rates before and after implementation of a multi-faceted quality improvement program (QIP) focused on advanced airway management (AAM).

## Methods

We designed a retrospective before and after cohort study to test the hypothesis that a multi-faceted AAM QIP improved the quality of paramedic intubation in our critical care transport organization, as measured by FPS and DASH-1A rates.

### Setting

Ornge is the sole critical care transport provider for Ontario, Canada, covering an area of just over one million km^2^ and a population of close to 15 million. Most of the population live in the Southernmost areas of the province, and access to critical or tertiary care requires often long transfers for the remote population in Northern Ontario. The organization operates six rotor wing bases, four land vehicle bases, two fixed wing bases and one base with access to rotor and fixed wing assets. In addition, Ornge provides education and clinical oversight for the Toronto Paramedic Services (TPS) critical care transport program. All aspects of call taking and dispatch are handled at one central operations control centre. Within Ornge and TPS, three levels of paramedics are trained to provide AAM.

- Advanced care paramedics (ACP-L) provide advanced life support (ALS) interventions including AAM and a limited number of intravenous (IV) medications such as Morphine.
- Advanced care flight paramedics (ACP-F) provide AAM including RSI but have some restrictions regarding higher-risk drugs such as Propofol or Epinephrine
- Critical care paramedics (CCP) provide a wide range of critical care interventions, including RSI, comparable to the level of care available in most emergency departments

Entry into the Ornge ACP-F or CCP programs requires provincial ACP-L certification. In Ontario, paramedics achieve ACP-L certification through successful completion of a two-year primary care paramedic (PCP) program followed by a one-year ACP-L college program. Upon acceptance to the Ornge ACP-F/CCP training program, paramedics complete a minimum of 18 months of theoretical modules, practical skill practice, simulation training, clinical rotations, and a certification exam. Airway management is an important aspect of this initial education and consists of a combination of theoretical, skill-based and simulation training, field-based evaluations, and certification exams.

### Intervention

The QIP was implemented across the organization in 2018 and 2019. It was led by clinically active paramedics with support from transport medicine physicians, respiratory therapists, and educational specialists. It included the purchase and distribution of new equipment, standardization of equipment and procedures, and provider training in these areas. Table 1 provides a comparison of advanced airway management in the organization before and after implementation of the airway management QIP.

**Table 1:** Advanced airway management in the time periods before and after a quality improvement program

	2016–2017	2020–2021
Annual training	Based on needs analysis No specific focus on advanced airway management	50% focus on advanced airway management Simulation and skill stations
Airway equipment	Direct laryngoscope AirTraq™ video laryngoscope King LT™ LMA™	CMAC™ video laryngoscope with standard geometry and hyper-angulated blades Direct laryngoscope iGel™
Standardization	Variable across the organization	Standardized contents and layout of paramedic response bags Standardized kit dump
Procedures	DFI encouraged Provider’s choice of intubation strategy push dose phenylephrine for hypotension	Checklist mandatory Apneic oxygenation RSI encouraged, Rocuronium for most patients, Succinylcholine also available VL and bougie as standard first attempt Push dose epinephrine and/or phenylephrine for hypotension

### Inclusion/exclusion criteria

We included all patients receiving AAM and where the initial AAM strategy was orotracheal intubation. We excluded cases where the initial AAM strategy was nasotracheal intubation or the placement of a supraglottic airway. We included pediatric patients and those in cardiac arrest.

### Data collection

Data was collected from the organization’s electronic records database containing all patient records. Patient demographics, intubation successes or failures, vital signs were extracted automatically. If data was missing, a manual chart review of the respective records was undertaken to complete data fields, where possible.

### Definitions

During the study period, an intubation attempt was defined as the insertion of a laryngoscope beyond the patient’s teeth or gums for the purpose of achieving tracheal intubation. Successful intubation required confirmation of end tidal carbon dioxide (EtCO2) for multiple breaths and/or confirmation of ETT placement on chest x-ray. For DASH-1A rates, we excluded patients being intubated during cardiac arrest. For all other patients, DASH-1A success was defined as intubation on the first attempt, systolic blood pressure (SBP) ≥ 90 mmHg and SpO2 ≥ 90% for 10 min following intubation. Of note, we included patients whose highest SBP or SpO2 prior to intubation were lower than the cut-off of 90 mmHg or 90%, respectively. For pediatric patients, the cut-off for SBP was adjusted to the lower margin of the 95% percentile bracket of the normal distribution for the patient’s age.

### Statistical analysis

Data were anonymized and statistical analysis performed using Stata Version 14 (StataCorp). We divided the study period into a pre-, peri-, and post-intervention period, covering the years 2016–2017, 2018–2019, and 2020–2021, respectively. We used the Chi-square and Kruskal–Wallis tests to examine for statistical significance of differences between the pre- and post-intervention periods for categorical and non-normal continuous data, respectively. *P*-values of < 0.05 were considered to have reached statistical significance.

### Ethics approval

The study was reviewed and approved by the Ornge Research and Scholarly Activities Committee and research ethics approval was granted by the University of Toronto (REB: 30628).

## Results

We identified 484 patient records of AAM with a first strategy of orotracheal intubation during the time period from January 2016 to December 2021. A total of 168 paramedics were involved in these cases as either intubator or airway assistant, with a median of eight cases per paramedic over the six-year study period (interquartile range [IQR] 4–12). Table 2 provides an overview of demographics and distributions of these cases.

**Table 2:** Characteristics of all cases with advanced airway management and oral endotracheal intubation as first strategy

Total patient episodes	484 (100%)
Year, n (5)	
2016	73 (15%)
2017	71 (15%)
2018	85 (18%)
2019	82 (17%)
2020	86 (18%)
2021	87 (18%)
Gender (male), n (%)	320 (66%)
Age, median (95% percentile)	50 (12—79)
Type of call, n (%)	
Inter-facility	246 (51%)
Modified scene	128 (26%)
Scene	110 (23%)
Diagnosis category n, (%)	
Trauma	160 (33%)
Trauma—vital signs absent	26 (5%)
Medical—neuro	91 (19%)
Medical—respiratory	54 (11%)
Medical—cardiac	31 (6%)
Medical—toxicology	21 (4%)
Medical—other	43 (9%)
Medical—vital signs absent	43 (9%)
Level of care, n (%)	
Critical care	368 (76%)
Advanced care (flight)	112 (23%)
Advanced care (land)	4 (1%)

**Fig. 1:**
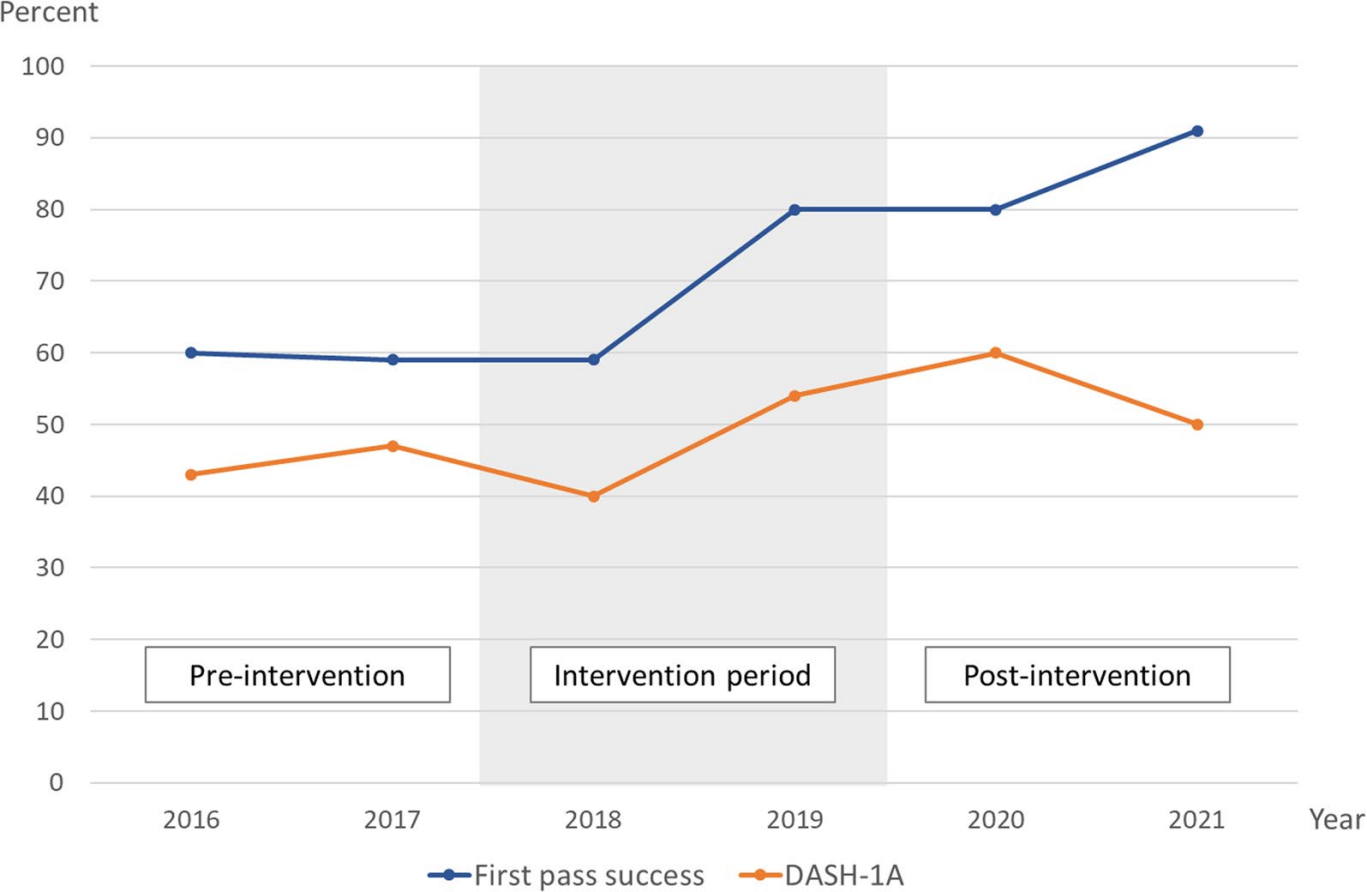
First pass success and DASH-1A rates by year

Overall, the first pass intubation success (FPS) rate was 72% (350/484). There was a significant increase in FPS from the pre-intervention period (60%, 86/144) to the post-intervention period (86%, 148/173), *p* < 0.001. 69 (14%) of patients were intubated during cardiac arrest, with a FPS rate of 62% (43/69). These 69 patients were excluded from DASH-1A success rate analyses. Of the remaining 415 patients, 205 (49%) were intubated on first attempt and without the occurrence of hypoxia or hypotension. Of the 210 patients without DASH-1A success, 61 patients (29%) had pre-existing hypotension or hypoxia below the SBP of 90 mmHg and SpO2 of 90% cut-off, respectively, prior to intubation. DASH-1A success rates improved from 45% (55/122) during the pre-intervention period to 55% (84/153) but this difference did not meet pre-defined statistical significance (*p* = 0.1). Figure 1 outlines the development of FPS and DASH-1A rates over the study period.

The use of VL for first intubation attempts increased from 0% (0/144) in the pre-intervention period to 88% (153/173) in the post-intervention period (*p* < 0.001). Table 3 provides an overview of factors which were hypothesised to be potentially associated with FPS and DASH-1A rates, based on previous literature.

**Table 3:** Explorative analysis of factors potentially associated with FPS and DASH-1A rates

	FPS rate (n = 484)		DASH-1A (n = 415)	
Overall	350/484 (72%)		205/415 (49%)	
Video laryngoscope				
First attempt with VL	149/174 (86%)	*p* < 0.001*	82/154 (53%)	*p* = 0.20
First attempt without VL	201/310 (65%)		123/261 (47%)	
Neuromuscular blockage				
Rapid sequence induction	151/191 (79%)	*p* = 0.02*	105/191 (55%)	*p* = 0.04*
Drug facilitated intubation	154/224 (69%)		100/224 (45%)	
* N/A (cardiac arrest)*	*45/69 (65%)*		*N/A*	
Base intubation volume				
High (> 10 per year)	193/264 (73%)	*p* = 0.50	121/238 (51%)	*p* = 0.60
Medium (10—5 per year)	114/164 (70%)		56/123 (46%)	
Low (< 5 per year)	43/56 (77%)		28/54 (52%)	
Type of call				
Scene	65/110 (59%)	*p* = 0.01*	17/62 (27%)	*p* = 0.001*
Modified scene	98/128 (77%)		60/112 (54%)	
Interfacility	187/246 (76%)		128/241 (53%)	
Level of care				
Critical care	274/368 (74%)	*p* = 0.06	157/315 (50%)	*p* = 0.7
Advanced care	76/116 (66%)		48/100 (53%)	

*Statistically significant at *p* < 0.05, VL = video laryngoscopy, FPS = first pass success, DASH-1A = Definitive Airway Sans Hypoxia/Hypotension on First Attempt

## Discussion

This retrospective database analysis demonstrated that a multi-faceted advanced airway management quality improvement program resulted in a significant improvement in first pass success rates of endotracheal intubation in the setting of paramedic-delivered critical care transport. DASH-1A rates improved to a lesser degree, and this change was not statistically significant. In an exploratory analysis, the use of paralytics as well as intubation in a healthcare facility (as opposed to intubation during scene calls) were associated with higher FPS and DASH-1A rates. The use of VL was associated with higher FPS rates but not higher DASH-1A rates.

We believe that the increase in FPS rate from 60 to 86% (for the pre- and post-intervention period, respectively) proves the effectiveness of our organization’s AAM QIP. While the post-intervention results are encouraging, they are also comparable to previously published FPS rates from other prehospital or retrieval services [10, 18–20]. Our explorative analysis suggests that the organizational direction from DFI towards RSI and the introduction of VL might have contributed to increasing FPS rates. Paralysis with neuromuscular blockers has been shown to increase FPS rates during intubations in intensive care units and emergency departments, when compared to DFI [21, 22]. To our knowledge, there is no research which directly compares intubation FPS rates with and without paralysis in the prehospital and retrieval setting. A randomized controlled trial comparing intubation with Midazolam and Etomidate, without paralytics in both groups, demonstrated a low overall intubation success rate of only 76% [23]. This contrasts with multiple large case series of prehospital RSIs, where overall intubation success rates trend towards 100% [12, 18, 20]. Prehospital RSI is the recommended strategy for prehospital intubations (other than in cardiac arrest or peri-arrest situations) by the Association of Anaesthetists of Great Britain and Ireland [2].

The best available evidence for the use of VL, and specifically the C-MAC video-laryngoscope used in our organization, is a randomized controlled trial from a German physician-staffed helicopter emergency medical service (HEMS) [24]. FPS rates in this service were 95% and 79% with VL and DL, respectively (*p* = 0.007). A final factor associated with FPS in our organization was the location in which AAM occurred. FPS rates were significantly lower when intubations were undertaken during scene calls. The majority of these cases were trauma patients and intubation usually occurred in the back of a land ambulance. The lower FPS rate is likely a reflection of the underlying severity and acuity of these cases, limited space around the patient, and the challenges of intubation of trauma patients, when compared to patients with medical critical illness inside a healthcare facility [1, 18].

For our second primary outcome, DASH-1A success rates, our data showed a non-statistically significant increase from 45 to 55% (pre- and post-intervention, respectively). While the use of paralytics and the type of call were associated with higher FPS and DASH-1a rates, the increased FPS rates seen with the use of VL did not translate into higher DASH-1A rates. It is worth noting that most of the interventions of the QIP, particularly the use of a bougie and VL, were aimed at overcoming anatomical challenges of intubation. While this process has led to higher FPS rates over time, it did not directly address some of the physiological challenges of AAM, in particular avoidance of hypoxia and hypotension [25]. This highlights the importance of clinical governance with ongoing monitoring and training for prehospital AAM programs [9, 17]. In our organization, the 2022 annual training program will include simulation specifically focused on physiologically difficult intubations, particularly pre-intubation resuscitation of the severely hypoxic, hypotensive, or acidotic patient [25].

An important challenge that our organization faces in assuring safety and excellence in AAM is the relatively infrequent occurrence of AAM. On average, our paramedics are only involved in little more than one case of AAM per year, with some paramedics only being involved in AAM approximately once every 5 years. While the exact number of intubations or AAM cases required to maintain competence after initial training continues to be debated, many high-performing HEMS provide in excess of 100 prehospital RSIs per base per year [12, 18, 26]. In contrast, almost half of intubations in our dataset were undertaken by paramedics working at bases with less than 10 intubations per year. As part of the AAM QIP, paramedics are now required to participate in a minimum of four to six simulated AAM scenarios per year. To further maximise learning from these infrequent cases, structured debriefs of all DFIs and RSIs as well as regular clinical governance meetings with presentation of AAM cases were introduced to our organization mid-2021.

### Limitations

This before and after study cannot clearly prove cause and effect of the AAM QIP and improved intubation FPS rates. However, the effect size is considerable and in keeping with previous literature. An important limitation is that the multiple aspects of the QIP were introduced in parallel, and due to the relative infrequence of intubations in our service, there is a likely delay between intervention and effect. As such, we were unable to clearly attribute changes in FPS or DASH-1A rate to individual interventions. Importantly, some of the changes made during the QIP, such as standardized equipment, the use of a pre-intubation checklist, or the use of a bougie, was not documented in individual patient charts. We were therefore unable to assess the effectiveness of these interventions individually. Finally, data completion and accuracy are well-documented challenges in retrospective database analyses. We undertook manual reviews of charts were required to minimise this issue, and the data entry and data retrieval process was the same for the pre- and post-intervention period.

## Conclusions

A multi-faceted AAM QIP resulted in statistically significant increase in intubation FPS rates and a non-significant improvement in DASH-1A rates. A combination of modern equipment, targeted training, standardization, and ongoing clinical governance is required to achieve and maintain safe intubation by paramedics in the prehospital and retrieval environment.

## Data Availability

Research ethics approval only included the use of data for this specific research project. As such, we are unable to share our data with other researchers.
